# A novel experimental platform to monitor solid/water interfaces under freeze–thaw cycles

**DOI:** 10.1111/jmi.70017

**Published:** 2025-08-05

**Authors:** Chiara Recalcati, Rossella Yivlialin, Lamberto Duò, Alberto Guadagnini, Gianlorenzo Bussetti

**Affiliations:** ^1^ Dipartimento di Ingegneria Civile e Ambientale Politecnico di Milano Milano Italy; ^2^ Dipartimento di Fisica Politecnico di Milano Milano Italy

**Keywords:** Atomic Force Microscopy, experimental set‐up design, Highly Oriented Pyrolytic Graphite, interfacial freezing dynamics, solid/liquid interface

## Abstract

We design and implement an original experimental platform resting on Atomic Force Microscopy (AFM) to capture nanoscale insights into key characteristics of solid/water interfaces subject to freeze–thaw conditions. The work is motivated by the observation that freezing and thawing underpin a variety of processes in the context of, e.g., climate and material sciences or cryobiology. Despite their key role, fundamental processes driving freezing and thawing are still elusive and their direct documentation is still challenging. This primarily stems from operational difficulties in replicating these processes under laboratory conditions, as well as constraints of current technology in matching temporal and spatial scales at which these phenomena take place. Here, we propose an experimental strategy to control freezing at solid/water interfaces while maintaining the bulk water as liquid. Our platform favors operational simplicity and can be integrated with any tip‐scanning AFM. The strength of our set‐up is assessed upon experiments performed on Highly Oriented Pyrolytic Graphite (HOPG) as a model substrate.

## 1 INTRODUCTION

Freezing and thawing processes taking place at solid/water interfaces are key in driving a variety of environmental and engineered scenarios. These include settings related to, e.g., climate^1^ and material^2^ sciences, or cryobiology.^3^ For example, nucleation of ice from water vapor onto mineral airborne dust in the upper atmosphere plays a critical role in regulating precipitation formation and influencing Earth's radiation balance.^4^, ^5^, ^6^, ^7^ Freeze–thaw cycles taking place at the interface between pore water and geomaterials that make up permafrost determine the fate of anthropogenic^8^, ^9^, ^10^ or biogeochemical^11^, ^12^ pollutants and carbon release^13^ in Arctic and Alpine environments experiencing dynamic climate changes. Rates of degradation of physical attributes (including mechanical properties) of building materials^14^ and rocks^15^ in severe environments are also influenced by the occurrence of freeze–thaw cycles. Having at our disposal the capability to accurately control ice nucleation dynamics is also at the basis of the optimization of cryopreservation strategies of biomaterials and can avoid the possibility of emergence of cryoinjuries to biosamples.^16^, ^17^

Despite their relevance across various contexts, significant knowledge gaps surrounding fundamental processes governing dynamics associated with freeze–thaw cycles at solid/liquid interfaces and their effects on the underlying substrate are still persisting. This is ultimately due to difficulties of mimicking the complex nature of interactions governing ice formation, evolution, and thawing under controlled laboratory settings. In this context, a variety of techniques have been proposed to investigate ice/vapor (Refs. 2, 18, 19 and references therein) and ice/solid (Ref. 20 and references therein) settings. Among these, Atomic Force Microscopy (AFM) is widely used to quantitatively assess mechanical and frictional surface properties^20^, ^21^, ^22^ and molecular‐ to nanoscale patterns.^23^, ^24^ Otherwise, even as solid/water interfaces subject to freeze–thaw processes are ubiquitously found across various scenarios (comprising, e.g. permafrost or snow dynamics), only a limited number of studies focus on these processes and the way their effects manifest at a fundamental level. In this context, Zepeda et al.^25^ rely on AFM and develop an original set‐up comprising a set of two chambers designed to control temperature and relative humidity. The internal chamber encapsulates the sample and the AFM scanner. Temperature therein is regulated through a combination of multiple Peltier junctions and cold nitrogen gas. The cold plates of the thermoelectric devices laterally bound the chamber. Copper heat exchangers are mounted on the hot plates. Heat is then dissipated through flux of nitrogen gas. The outer chamber is built around the inner one and is tailored with apertures that allocate the optical microscope and the AFM base and must be properly sealed. Here, temperature is regulated only through nitrogen flux. This set‐up enables accurate investigation of both ice/water and ice/vapor interfaces. Otherwise, its proper operability requires cumbersome devices that must be customized to the specific AFM configuration. These, along with practical difficulties in conveying samples to the AFM stage through multiple chambers, limit the flexibility of their system. A set‐up enabling investigation of ice/water interfaces has been recently developed by Chasnitsky et al.^26^ These authors utilize a custom‐built cold finger device, consisting of a copper pyramid encased in a mica disk with a drilled hole. A liquid cell is secured to the mica sheet. The system is then mounted on two superimposed Peltier junctions. Localized supercooling of the solution is induced only in close proximity of the finger device and enables to obtain local freezing conditions. The bulk solution is otherwise liquid. While this set‐up enables investigation of ice/water interfaces, its applicability to investigate ice nucleation from liquid environments on other substrates such as, e.g., minerals, is still unclear as the finger device must be constituted by a highly thermally conductive material.

Here, we design and develop an original experimental platform integrating AFM imaging and accurate temperature control. It is specifically designed to allow for simplicity and flexibility in imaging of freeze–thaw dynamics in liquid environments. In contrast to existing experimental strategies, integration of our platform to any tip‐scanning AFM is straightforward and solely requires customization of a heat dissipation sample plate. It offers a flexible and versatile technical solution to investigate any solid/water interface (i) under stable thermal conditions and/or (ii) subject to repeated freeze–thaw cycles. It does not require a specifically tailored sample preparation. Reliability and operational simplicity of our novel experimental platform are assessed upon relying on Highly Oriented Pyrolytic Graphite (HOPG). This is selected as a model substrate due to its flatness, stability and chemical inertness in water. The remainder of the study is structured as follows. Section 2 illustrates key elements of our original experimental set‐up and protocols. Section 3 illustrates the use of the designed set‐up to image the surface of a HOPG sample subject to multiple freeze–thaw dynamic cycles. Section 4 outlines our main conclusions.

## 2 EXPERIMENTAL METHODS

**FIGURE 1:**
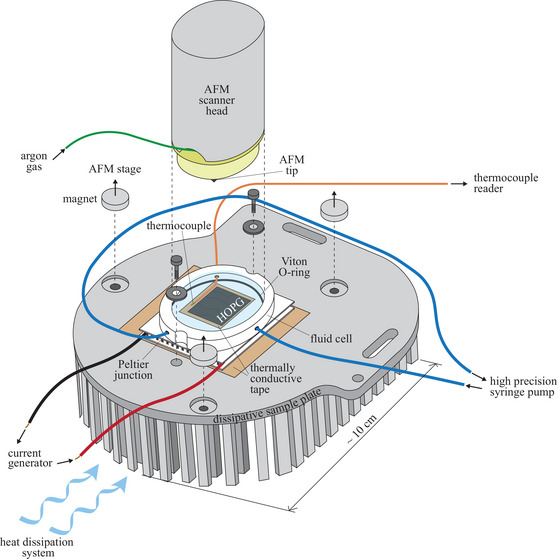
Sketch of the designed experimental set‐up integrating AFM technology with thermal regulation and monitoring tools.

Our set‐up provides an innovative integration of AFM imaging technology (Keysight 5500) with thermal regulation and monitoring tools enabling accurate control over ice formation at any solid/water interfaces. A sketch of the experimental set‐up is depicted in Figure 1.

The designed experimental platform comprises a (i) thermal regulation system, (ii) a heat dissipation system and (iii) a flow regulation device. The thermal regulation system relies upon a Peltier junction connected to a power supply. This configuration yields refined control over temperature at the solid/water interface, the associated experimental uncertainty being approximately $\pm 0.1^{\circ }C$. Temperature is continuously monitored throughout the experiment via a Chromel–Alumel thermocouple. The latter is secured to the Peltier junction via a thermally conductive (copper) adhesive tape. Readings from the thermocouple document that the cold plate of the Peltier junction attains thermal stability within ∼1 min after tuning the supplied voltage. Maintaining localized cooling requires dissipation of the heat produced at the junction hot plate. We ensure effective functioning of the Peltier junction upon design and optimization of a heat dissipation system. The latter encompasses a custom‐designed aluminium sample holder (equipped with a set of protrusions, as depicted in Figure 1) onto which the Peltier junction is affixed through conductive adhesive tape. The sample holder is magnetically attached to the AFM sample plate and is specifically tailored for compatibility with the commercial Keysight 5500 system. This, in turn, enables seamless integration with our imaging technology. Depending on the degree (and rate) of cooling required, thermal dissipation can be further enhanced through, e.g., (i) an air‐cooled fan, (ii) a water‐cooling system or (iii) liquid nitrogen vapors. Having at our disposal a collection of options provides flexibility in experimental design, accommodating a wide range of cooling requirements. In this study, we enhance thermal dissipation through liquid nitrogen vapors. This choice minimizes mechanical vibrations of the AFM infrastructure. The sample under investigation is placed on the cold surface of the Peltier junction. A Teflon cell (Volume ≈2.5 mL) open to air is screwed to the sample holder and sealed with a Viton O‐ring. This setting effectively prevents fluid leakage and maintains integrity of the whole set‐up. The cell is connected through piping (Tygon tubing, 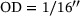, 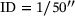) to a high precision syringe pump (Hardard Apparatus, 33 Dual Drive System). The latter is equipped with two independent modules and is filled with ultra‐pure Milli‐Q water (18.2MΩ cm). The flow regulation system allows maintaining the bulk water at room temperature, thus minimizing interference of larger‐scale thermal effects. This enables one to focus on critical interfacial phenomena upon confining the freezing process to fluid layers that are in close proximity to the sample surface. The AFM scanner head is designed to work in liquid environments. Yet, operating at temperatures below $10^{\circ }C$ yields condensation of water droplets on the viewport of the AFM nose cone. These interfere with the AFM laser reflection path and constitute a severe challenge to imaging procedures. We circumvent this issue upon delivering a continuous flow of argon (Ar) gas through a thin tube integrated into the scanner head. Condensation‐free operations ensure uninterrupted imaging and enhance the overall quality of collected data. The AFM apparatus employed in this work is compatible with contact as well as non‐contact modes. As such, it offers exceptional imaging flexibility. It is otherwise noted that non‐contact mode operations could be hindered by (even minor) discrepancies in the grounding of electronic components associated with the temperature regulation system. This is then prone to inducing a 50 Hz signal into the system, which, in turn, can hamper the acquisition of high‐quality topographic data. Here, we rely on non‐contact imaging and avoid this issue upon meticulously grounding all electronic components to the AFM apparatus. We employ silicon tips with an aluminium‐coated cantilever (Bruker, TESPA‐V2, spring constant $k=37\;{\mathrm{Nm}}^{-1}$). In addition to its primary function of aligning the laser beam onto the cantilever, the AFM camera is also employed to observe real‐time formation and melting of the ice layer on the sample surface. As such, it offers an additional element of visualization and control of freeze–thaw dynamics. Further to this, relying on such strategy offers an added value in the presence of solutions with diverse chemical composition which can alter the freezing point. As a model substrate, we consider a z‐grade HOPG supplied by Optigraph. This material offers (i) an atomically flat surface, (ii) exceptional chemical inertness and (iii) superior compatibility with high‐resolution imaging techniques. As such, it can be used to assess reliability of the designed experimental set‐up. The HOPG is prepared by exfoliation using a thermally conductive adhesive tape. The freshly exfoliated sample, composed of multiple graphite layers, is then mounted in the Teflon cell and affixed onto the cold surface of the Peltier junction.

## 3 RESULTS AND DISCUSSION

We provide a proof‐of‐concept of the viability of our experimental approach and implemented assemblage of technologies upon investigating two diverse settings. These also enable us to assess the way the sample preparation protocol might impact on HOPG morphology changes due to repeated thermal stresses induced by freeze–thaw cycles. In *Setting A*, we subject a freshly exfoliated HOPG sample to five repeated freeze–thaw cycles. Prior to the freezing step of each freeze–thaw cycle, the AFM probe is lifted from the surface by 120 μm. This enables us to avoid damaging of the tip and to minimize the possibility of any heat transfer from the AFM cantilever to the sample surface. Freezing is then achieved upon instantaneously lowering temperature to $-2^{\circ }C$. Thawing is otherwise designed upon inducing an instantaneous increase of the temperature up to values around $15^{\circ }C$. We image a (10×10) $\mu m^{2}$ portion of the HOPG sample prior to the beginning of a given freeze–thaw cycle and after thawing is attained. Otherwise, in *Setting B* we first leave the sample in contact with (static) Milli‐Q water for a time >2 h prior to inducing freezing. In this setting, each freeze–thaw cycle is designed to span temporal windows of the order of a few minutes.

**FIGURE 2:**
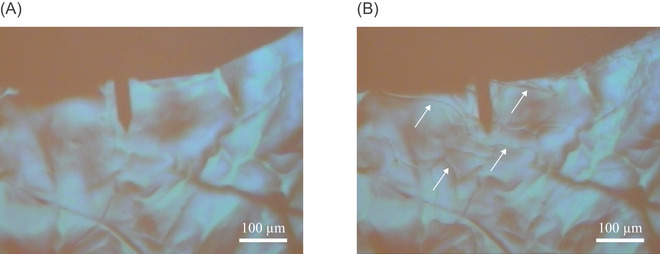
Snapshots taken by the AFM camera (A) at room temperature ($T=25^{\circ }C$) and (B) during the first freezing cycle of the experimental procedure. White arrows in (B) highlight ice subdomain edges, whose presence documents the formation of the ice layer on top of the HOPG surface.

Freezing of the water in contact with the substrate and subsequent ice formation are assessed through (i) imaging via the AFM camera and (ii) tracking the laser sum signal resulting from the reflection of the laser spot on the AFM detector. Figure 2A,B depicts snapshots of the freshly exfoliated HOPG sample (Setting A) at room temperature ($T=25^{\circ }C$) and during the first freezing cycle, respectively. While the exact identification of the speed of propagation of the freezing front across the surface is fraught with uncertainty, direct observation from the AFM camera documents that freezing takes place almost instantaneously at a temperature between $0^{\circ }C$ and $-2^{\circ }C$. This corresponds to ice formation at relatively moderate supercooling conditions (compared to nucleation temperatures reported by, e.g. Chasnitsky et al.^26^). We note that thermal gradients between the bulk water and the HOPG surface could yield an overestimation of the actual nucleation temperature detected via our thermocouple. Our observations show that the thin ice layer is organized across diverse subdomains. These are clearly visible in Figure 2B (white arrows highlighting internal boundaries between subdomains). From a qualitative standpoint, one can recognize that the presence of edges demarcating diverse subdomains is consistent with observations documented by Chasnitsky et al.^26^ These elements then vanish as soon as the sample temperature is increased above $0^{\circ }C$. The direct visualization of freezing and thawing documented through the AFM camera imbues us with confidence about our ability to effectively control temperature at the solid/water interface. It also enables us to accurately quantify the time interval during which the surface remains covered by ice. As a further element, we note that the distinct refractive properties of ice with respect to water imprint the laser path. In turn, this yields a misalignment of the laser spot on the detector, thus providing additional proof of ice formation on the surface. Upon thawing, the reflected laser spot naturally realigns on the AFM detector.

**FIGURE 3:**
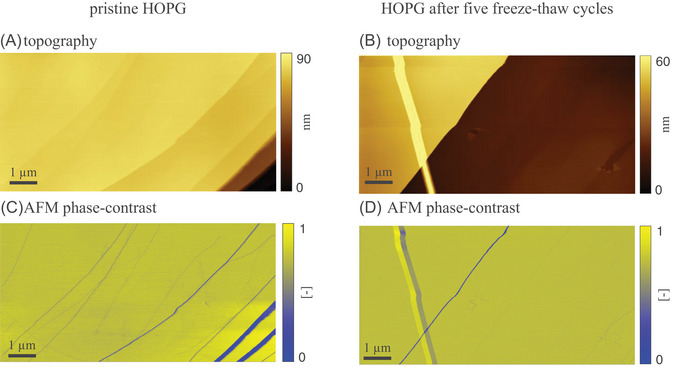
AFM images of freshly exfoliated HOPG (Setting A) taken (A, C) at the beginning of the experiment at $T=25^{\circ }C$ and (B, D) after five subsequent freeze–thaw cycles at $T=15^{\circ }C$.

Morphological traits of HOPG are systematically investigated before and after ice formation on the solid/water interface. Figure 3 illustrates AFM topography and phase‐contrast signals associated with Setting A and taken at ambient conditions right after contact of the HOPG sample with Milli‐Q water (room temperature, $T=25^{\circ }C$; panels A and C) and at the end of the fifth freeze–thaw cycle (corresponding to $T=15^{\circ }C$; panels B and D). The stability of the pristine HOPG surface at room temperature and the high quality of the sample are evidenced by the surface topography (Figure 3A) and the AFM phase‐contrast image (Figure 3C). To facilitate direct comparison of results, all AFM phase‐contrast signals included in the paper are depicted as normalized values. Figure 3A,C shows uniform flat terraces and steps of various height. Images acquired at the end of the experiment (Figure 3B,D) starkly resemble those associated with the pristine HOPG. The same behavior is documented also across intermediate cycles. This finding confirms the remarkable robustness and structural integrity of the freshly exfoliated HOPG surface under the experimental conditions at which it is subject. They further sustain its suitability as a model substrate for studies involving repeated phase transitions of water. Figure 3B,D also documents that AFM imaging remains free from drift even as the sample is subject to temperature changes, the surface morphology showing no signs of degradation or damage. The stripe feature that can be observed on the left side of Figure 3B,D does not affect the overall quality of the experiment. In addition, the high imaging quality of Figure 3B,D documents that neither the AFM cantilever nor the tip are damaged during freezing. This imbues us with confidence about the ability of our experimental set‐up to sustain repeated actions of freeze–thaw cycles.

**FIGURE 4:**
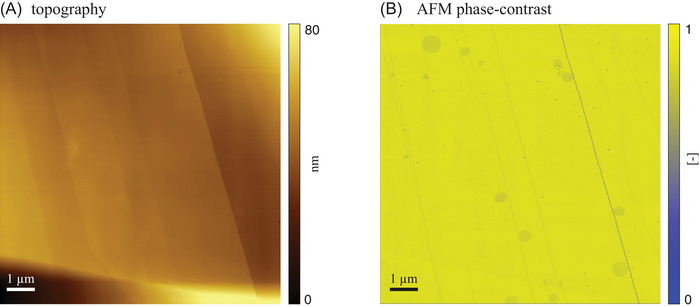
AFM (A) topography and (B) phase‐contrast images of a HOPG sample (Setting B) immersed in water prior to freeze–thaw cycles at $T=25^{\circ }C$.

Figure 4 depicts AFM topography and phase‐contrast images of an HOPG sample associated with our experimental Setting B prior to freeze–thaw cycles. The surface topography (Figure 4A) exhibits well‐defined steps of varying heights. Several (approximately circular) features can be detected upon close examination. We note that these features are barely detectable on the topography image (Figure 4A) while being starkly identifiable in its AFM phase‐contrast counterpart (Figure 4B). These features appear as markedly distinct from the expected flat and uniform appearance of the HOPG basal plane. The appearance of such features is consistent with experimental observations documented by Fang et al.^27^ These authors observe these geometrical shapes in the proximity of step edges while investigating nanobubble nucleation at the HOPG/air‐supersaturated water interface. These features are circular gas‐rich layers onto which interfacial nanobubbles can then grow and are identified by Fang et al. as wetting layers. The formation of such wetting layers is consistent with the type of water (i.e., Milli‐Q water) and the temporal observation window considered in our settings.

**FIGURE 5:**
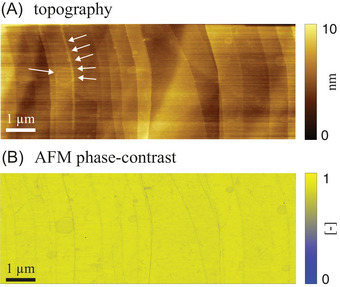
AFM (A) topography and (B) phase‐contrast images of a HOPG sample imaged relying on Setting B at $T=0.5^{\circ }C$.

Figure 5 depicts images of the system acquired at conditions corresponding to $T=0.5^{\circ }C$. The surface morphology (Figure 5A) reveals nearly uniform terraces, though the image appears to be slightly influenced by an initial drift during scanning (here scanning progresses from the top of the image). In this case, the presence of circular features is clearly documented in the AFM phase‐contrast signal (Figure 5B), similar to what we observe in Figure 4B. This finding provides experimental evidence of the stability of wetting layers also in the presence of cyclic freeze–thaw conditions. To the best of our knowledge, this is the first time that the presence of such circular wetting layers at the graphite/water interface is observed close to water freezing conditions. Visual inspection of Figures 4 and 5 suggests that the AFM phase‐contrast signal is spatially uniform or heterogeneous across terraces at room temperature or close to freezing conditions, respectively. This, in turn, suggests the presence of a spatially heterogeneous strength of the interaction between the substrate and the AFM tip close to freezing conditions. We observe local changes in the thickness of some step edges (white arrows in Figure 5A) and around the border of the circular wetting layer in the surface topography only when temperature approaches freezing conditions. Further targeted investigations are needed to unravel the mechanisms governing the evolution of these features and the ensuing implications on the dynamics of HOPG/water interfaces.

**FIGURE 6:**
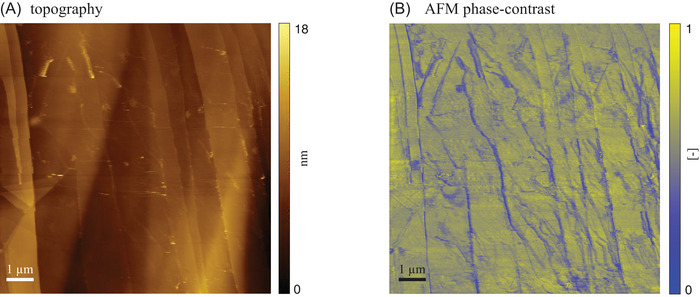
AFM (A) topography and (B) phase‐contrast of the HOPG sample imaged at $T=15^{\circ }C$ according to Setting B after five freeze–thaw cycles.

Figure 6 depicts the HOPG surface morphology imaged at $T=15^{\circ }C$ after five freeze–thaw cycles performed according to Setting B. While HOPG steps are clearly visible and similar to the pattern depicted in Figure 4, the sample morphology (Figure 6A) exhibits small clusters of varying size scattered across the HOPG basal plane and along some step edges. The presence of these elements is further emphasized in the AFM phase‐contrast signal (Figure 6B). Otherwise, circular features observed at room temperature (Figure 4) and at T = 0.5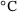 (Figure 5) are no longer detected. In addition, Figure 6 documents a general degradation in the quality of HOPG terraces after several hours of experimental observation under repeated freeze–thaw cycles.

## 4 CONCLUSIONS

We design and implement a novel experimental platform enabling direct in situ observation of any type of solid/water interfaces subject to repeated freeze–thaw cycles, including, e.g., metals, semiconductors, and minerals. We integrate AFM with temperature and flow regulation systems. The latter are aptly designed to enable accurate control over key environmental parameters (such as temperature at the interface and fluid flow velocity) while maintaining the bulk solution as liquid. The flexibility and potential of the set‐up are assessed upon two exemplary experiments (denoted as Setting A and B, respectively, in Section 3) involving imaging of an HOPG sample subject to freeze–thaw cycles. We then illustrate the way sample preparation protocols impact on HOPG behavior under repeated thermal stresses.

Key conclusions stemming from our work are summarized in the following:

- Our experimental set‐up maintains a temperature control accuracy of $∼\;0.1^{\circ }C$ using only a single thermoelectric device paired with a heat dissipation system. Our solution significantly reduces the technical complexity of existing experimental strategies that rely on multiple nested chambers.^25^ In addition, it enables effortless integration with any tip‐scanning AFM, solely requiring a customized sample plate. This versatility allows for the investigation of various substrates, opening new research opportunities in fields such as material and environmental sciences.
- Images of a freshly exfoliated HOPG sample (Setting A in Section 3; Figure 3) collected between five repeated freeze–thaw cycles evidence the presence of flat and uniform terraces and steps that remain unaltered by the thermal stresses. These surface patterns are consistent with the expected morphology of the HOPG basal plane and document the reliability of our experimental set‐up. Otherwise, circular features located near graphite step edges are observed on HOPG samples exposed to static water prior to the beginning of freeze–thaw cycles (Setting B in Section 3; Figures 4, 5 and 6). These are consistent with wetting layers that are precursors of nanobubble nucleating at graphite/water interfaces.^27^ When exposed to repeated freeze–thaw cycles, edges associated with these features exhibit localized thickening. Systematic investigation of this behavior will be the focus of future studies.
